# Elongating under Stress

**DOI:** 10.4061/2011/326286

**Published:** 2011-09-07

**Authors:** Eulàlia de Nadal, Francesc Posas

**Affiliations:** Cell Signaling Unit, Departament de Ciències Experimentals i de la Salut, Universitat Pompeu Fabra (UPF), C/ Doctor Aiguader 88, 08003 Barcelona, Spain

## Abstract

In response to extracellular stimuli, mitogen-activated protein kinases (MAPKs) modulate gene expression to maximize cell survival. Exposure of yeast to high osmolarity results in activation of the p38-related MAPK Hog1, which plays a key role in reprogramming the gene expression pattern required for cell survival upon osmostress. Hog1 not only regulates initiation but also modulates other steps of the transcription process. Recent work indicates that other yeast signalling MAPKs such as Mpk1 modulate transcriptional elongation in response to cell wall stress. Similarly, mammalian MAPKs have also been found associated to coding regions of stress-responsive genes. In this paper, significant progress in MAPK-regulated events that occur during the transcriptional elongation step is summarized, and future directions are discussed. We expect that the principles learned from these studies will provide a new understanding of the regulation of gene expression by signalling kinases.

## 1. Introduction

Signal transduction pathways allow cells to sense and respond to extracellular stimuli. MAPK modules are conserved signalling elements utilized in many intracellular signal transduction pathways in eukaryotic organisms [1]. Each MAPK module is activated by specific types of stimuli to induce specific adaptive response. The central core of MAPK systems consists of a tier of kinases, a MAPK kinase kinase (MAPKKK) that phosphorylates and activates a MAPK kinase (MAPKK) on serine and threonine that in turn phosphorylates the MAPK on a threonine (sometimes serine) and tyrosine residue. In yeast *Saccharomyces cerevisiae,* there are several MAPK cascades (for reviews of function and regulation in yeast MAPK signalling, see [2–8]).

**Figure 1:**
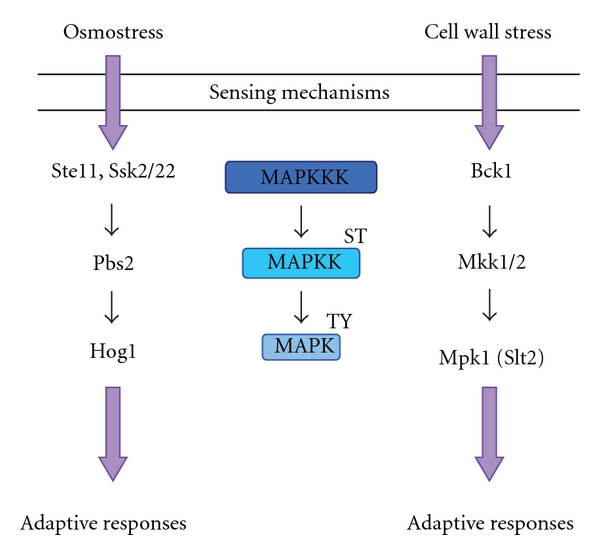
The HOG and CWI pathways. CWI signalling pathway is initiated at the plasma membrane through different sensing mechanisms and is activated by Pkc1 upon different cell wall stresses. The linear cascade consists in the Bck1 MAPKKK, which activates a pair of redundant MAPKK (Mkk1 and Mkk2) that in turn activates the Mpk1/Stl2 MAPK. In the HOG pathway, two independent upstream osmosensing mechanisms lead to the activation of the MAPKKK Ste11 and Ssk2/22. The Pbs2 MAPKK integrates both signals and activates the Hog1 MAPK. Both the HOG and CWI pathways are involved in the regulation of transcriptional elongation by specific types of stimuli to induce specific adaptive response.

 The HOG (high osmolarity glycerol) pathway is activated in response to osmostress by two upstream independent branches that converge on the MAPKK Pbs2, which controls Hog1 MAPK activity (see Figure 1) [2, 9–11]. Upon stress, Hog1 translocated into the nucleus [12, 13]. The CWI (cell wall integrity) pathway, which is comprised of Bck1, Mkk1/2, and the Mpk1/Slt2 (and its pseudokinase paralogue Mlp1) MAPK, becomes activated under a number of different conditions that compromise the structure and function of the yeast cell wall, including elevated growth temperature, pheromone-induced morphogenesis, and chemical cell wall antagonist (see Figure 1) [5, 14]. MAPK pathways are known to be conserved during the evolution of the entire eukaryotic kingdom. The Hog1 functional ortholog in mammalian cells is the p38 family of stress-activated MAPKs (SAPKs) and responds to several stresses [15, 16]. Mpk1 is a functional homolog of human Erk5 (for extracellular signal-regulated kinase), which is activated in response to growth factors as well as various stresses [17]. 

In yeast, these two MAPKs play an important role in controlling gene expression, and they both modulate transcription initiation and elongation steps of the transcription cycle. We will focus here on the role of Hog1 and Mpk1 in regulation of transcription elongation.

## 2. An Overview of Regulation of Transcriptional Initiation by Hog1 and Mpk1

Among other functions, Hog1 is a master regulator of reprogramming gene expression in response to osmostress. Upon stress, the yeast genome alters its expression pattern up to 20% depending on the strength and duration of the stress [18–24]. A major part of these changes are regulated by Hog1 through several unrelated transcription factors such as Msn2/4, Hot1, Smp1, or Sko1, which work in combination at the specific stress-dependent promoters [19, 21, 25–28]. Recently, it has been reported the dynamics of binding of these transcription factors to their target genes in response to osmostress [19, 25]. The integration of this analysis with gene expression patterns reveals a complex dynamic and hierarchical network in which specific combinations of transcription factors activate distinct sets of genes at discrete times to coordinate a rapid and transient stress-adaptive response [25]. It is well known that, when transcription is initiated in response to osmostress, the MAPK is recruited to the osmoresponsive genes by specific transcription factors [26, 27, 29–31] and directly phosphorylates some of them [32–34]. Once bound to chromatin, Hog1 serves as a platform to recruit the RNA Pol II [32] and associated general transcription factors such as the Mediator or SAGA [30, 35] as well as histone-modifying factors [36]. It is worth noting that Hog1 is not the unique kinase that binds to chromatin. Actually, most MAPKs in yeast associate with genes that are their targets of transcriptional control [27, 37]. Further details in the regulation of transcriptional initiation by MAPKs and their implications for understanding control of gene expression are described in [38–40]. 

In response to cell wall stress, two known transcription factors, Rlm1 and SBF (Swi4 and Swi6), are controlled by Mpk1 by different mechanisms. Whereas Rlm1 is activated through direct phosphorylation [41–44], SBF is activated by a noncatalytic mechanism [37, 45–47]. Swi4 forms a complex with Mpk1 upon stress, and it associates with SBF-binding sites in the promoters of cell wall stress target genes [48]. Moreover, Mpk1 regulates Swi6 nucleocytoplasmic shuttling in a biphasic manner: first, formation of the Mpk1-Swi4 complex recruits Swi6 to the nucleus for transcriptional activation and, second, Mpk1 negatively regulates Swi6 by phosphorylation, which inhibits nuclear entry [49, 50].

## 3. The Hog1 MAPK Regulates Transcriptional Elongation

The transcription cycle consists of several steps, and elongation is a critical phase of transcription susceptible of strong regulation [51–53]. In addition to its association to promoters, Hog1 is also present at coding regions of stress-responsive genes, suggesting to have a more general role as chromatin-associated enzyme than previously expected. Actually, genome-wide chromatin binding of the MAPK has revealed that Hog1 is recruited to most of the transcribed regions of osmoinducible genes [26, 27]. Moreover, it is recruited to transcribed regions independently of the promoter bound-specific transcription factors dedicated to osmostress adaptation. By uncoupling Hog1-dependent transcription initiation from transcription elongation, it has been demonstrated that binding of Hog1 to stress-responsive coding regions depends on the 3′UTR regions. However, how SAPK is recruited to these specific 3′regions in response to stress remains so far unknown [31]. 

Which are the tasks of Hog1 at coding regions is still an open question. Recruitment of the kinase to the open reading frames (ORFs) is essential for an increased association of RNA Pol II and proper mRNA production in response to osmostress [31]. Moreover, Hog1 interacts with elongating RNA Pol II (phosphorylated at serine 2 and 5 of the C-terminal domain) as well as with general components of the transcription elongation complex. It is worth noting that the catalytic activity of Hog1 is required both for its binding to chromatin and to stimulate mRNA production during the elongation process. However, the identification of phosphorylation events mediated by the MAPK during transcription elongation remains open.

Binding of Hog1 to the stress-responsive ORFs is restricted temporally. Although the initial recruitment of the MAPK and RNA Pol II is similar, Pol II association is observed for a longer period upon osmostress, whereas binding of Hog1 is restricted at the initial phase of elongation [31]. This suggests a role for the MAPK at early stages of the elongation process. Chromatin structure is tightly regulated through multiple mechanisms, including chromatin remodelling, histone variant incorporation, histone eviction, and histone modification [54–56]. Actually, several genome-wide studies found a significant loss of histones from the promoter and coding regions of heavily transcribed genes throughout the genome [57]. 

Nucleosome positioning of stress-responsive loci is altered dramatically in a Hog1 MAPK-dependent manner during osmostress [58]. Hog1 physically interacts with the RSC chromatin remodelling complex to direct its association with the coding regions of osmoresponsive genes and allow for nucleosome rearrangements during transcriptional elongation upon stress. In the RSC mutants, RNA Pol II accumulates on stress-dependent promoters but not in coding regions. Moreover, the RSC mutants display reduced stress gene expression and enhanced sensitivity to osmostress [58]. Other chromatin remodelling complexes such as INO80 associated with the ORFs of stress genes in a stress-specific way [59, 60]. Mutants defective in subunits of the INO80 complex, as well as in several histone chaperone systems, lead to globally increased transcript levels upon osmostress and delayed repositioning of histones in ORF regions of stress genes. Thus, it seems that INO80 is relevant for the efficient downregulation of stress genes under acute stress conditions.

Single-cell experiments have shown that Hog1 nuclear accumulation increases linearly with stimulus. However, at low stress levels, the transcriptional output shows two distinct subpopulations, one responding and the other one not. Of note, this bimodality is reflected in chromatin remodelling and depends on both the retention time and concentration of Hog1 in the nucleus [61]. Thus, chromatin dynamics, together with transient MAPK activation, determines a transcriptional threshold in response to linear increase in signalling upon stress.

## 4. The Mpk1 MAPK Serves in Transcription Elongation

The role of MAPK in the modulation of transcriptional elongation is not restricted to Hog1. Other yeast signalling kinases, such as Fus3, PKA, or Mpk1, have been reported to associate to coding regions of activated genes [62]. Mpk1 associates with the coding region of the *FKS2* gene in response to cell wall stress although such binding does not require MAPK kinase activity [49]. This diverges from Hog1, where catalytic activity is essential for kinase recruitment.

How does Mpk1 associate with the *FKS2* coding region during transcription? Mpk1 is tethered to the elongation complex through its interaction with RNA Pol II-associated complex Paf1C complex [49, 63, 64]. Paf1 subunit interacts directly with Mpk1 through its docking motif (D-motif) in a cell wall stress-dependent manner. Interaction between both proteins requires the Swi4 and Swi6 but not Rlm1 transcription initiation factor. The *paf1-4A* mutant, which is unable to interact with Mpk1, is deficient for transcription elongation of the *FKS2* gene and renders cells sensitive to cell wall stress. Actually, in this mutant, Mpk1 is recruited to the promoter but does not progress into the coding region of *FKS2* in response to stress [49]. This is again a different scenario when compared to Hog1 and osmostress, since Mpk1 is moving from the initiation to the elongation complexes using the Paf1 complex as a scaffold. Of note, Paf1 and Hog1 are able to coimmunoprecipitate in response to osmostress [31] if such interaction is through the D-motif in Paf1 remains to be elucidated. A large number of short sense transcripts across the *FKS2* promoter-proximal region terminate under noninducing conditions [65, 66]. The critical function of the Mpk1-Paf1 association in transcription elongation is to prevent such premature termination under inducing conditions. Indeed, Mpk1-Paf1 interaction blocks recruitment of the Sen1-Nrd1-Nab3 termination complex to allow effective elongation of cell-wall stress genes [49]. Therefore, it is becoming increasingly apparent that yeast MAPKs play a key role in regulation of transcriptional elongation in response to cellular stress although the molecular mechanisms involved differ among kinases.

## 5. MAPK Signalling and Transcriptional Elongation in Higher Eukaryotes

Binding of signalling kinases to chromatin has been now shown in organisms other than yeast. Several reports support an essential role of p38 MAPK in the regulation of transcription upon inflammation and stress responses [67, 68] as well as during cell growth and differentiation [69–71]. In response to stress, p38 associates to chromatin as Hog1 does in yeast and allows for recruitment of RNA Pol II and transcriptional initiation. Similarly, anchoring of active p38 to target stress-dependent promoters is mediated by specific transcription factors [72]. Moreover, p38 is also present at coding regions depending on its activity upon stress, clearly suggesting that it might be travelling along with the nascent mRNA elongating machinery in a similar way as described in yeast [72]. Actually, it is described that the MAPK interacts with the RNA Pol II in mammalian cells [32]. 

p38 controls skeletal muscle differentiation by regulating the sequential activation of myogenic regulatory factors and their transcriptional coactivators, including chromatin remodelling enzymes (reviewed in [73]). However, whether the MAPK has a specific role during transcriptional elongation in muscle differentiation remains to be determined. 

The extracellular signal-regulated kinase (ERK) pathway also regulates gene expression. Erk1 is activated by progesterone and phosphorylates the progesterone receptor. Then, a complex of activated progesterone receptor, Erk1, and its target kinase Msk1 is recruited to the target promoters, where Msk1 phosphorylates histone H3 at serine 10 promoting chromatin remodelling and gene regulation (reviewed in [74, 75]). Once more, the specific role of the Erk1 kinase in transcriptional elongation in response to progestins remains to be elucidated. 

When expressed in yeast, the human Erk5 MAPK is activated in response to cell wall stress and suppresses the phenotypic defects of a *mpk1* mutant. Moreover, Erk5 is able to activate gene expression through Rlm1 as well as SBF transcription factors [17, 37]. There are evidences suggesting that MAPK regulation of Paf1C function is conserved in humans. Actually, Erk5 can interact with a predicted D-motif in the human PD2/PAF1 within the same region as that found in yeast Paf1 to drive transcription elongation [47].

Therefore, it is likely that MAPK regulation of transcription in higher eukaryotes is not restricted to initiation but also to elongation upon stress and that MAPK-driven mechanisms are conserved among eukaryotic cells.

## 6. Conclusions

**Figure 2:**
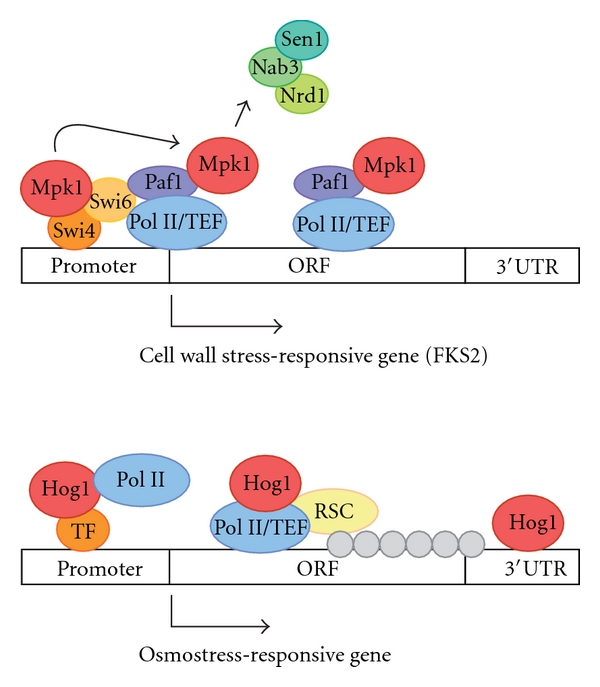
Regulation of transcriptional elongation by MAPK signalling pathways. Mpk1 and Hog1 MAPKs are key regulators of transcription in response to specific stresses. Whereas activated Mpk1 is moving from initiation to elongation complexes through its interaction with Paf1C complex, binding of Hog1 to the ORFs seems to be dependent on the 3′UTR region of the osmoresponsive gene. Association of Mpk1 with Paf1 serves as an antitermination factor by blocking recruitment of the Sen1-Nrd1-Nab3 termination complex to the cell wall stress-responsive gene. Hog1 acts as a stress-specific transcription elongation factor (TEF) and targets selectively the RSC complex to osmoinducible ORFs, which displaces nucleosomes contributing to the efficient activation of transcription.

Proper adaptation to stress is critical for cell survival. There has been a number of signalling networks involved in stress signal transduction, and, amongst them, MAPK signalling networks stand out. One important piece of the different adaptive strategies consists of a massive reorganization of the gene expression capacity. Protein kinases not only phosphorylate target proteins, but also themselves become part of the transcriptional complex. This suggests a new scenario in which signalling kinases, rather than simply relaying the signal to the transcriptional effectors, may function as integral components of the transcriptional complexes that activate expression of distinct target genes (see Figure 2). Actually, several recent reports have shown that the role of MAPK in the regulation of the transcription cycle is not limited to transcription initiation but rather extends to the process of transcriptional elongation. For instance, the Hog1 MAPK fits with the description of a bona fide elongation factor, with the feature that its role in elongation is restricted to osmoresponsive genes, and it has a pivotal role in nucleosome remodelling upon stress. Moreover, other MAPKs such as Mpk1 block premature transcription termination of stress-induced genes. Further studies are required to establish whether the mechanisms by which Hog1 and Mpk1 regulate transcriptional elongation are conserved in both MAPKs.

We are just beginning to understand the overlapping link between signal transduction pathways and modulation of transcriptional elongation. The molecular mechanisms of such relationship are far from fully being understood. For example, one unresolved question is how the MAPK Hog1 is recruited specifically to the osmo-responsive coding regions. Is there some special feature in the 3′UTR region of stress-dependent genes targeting the kinase? or is there some transcription elongation factor that specifically recruits the kinase? Another key question remaining in the field is which are the phosphorylation events mediated by MAPK on the transcriptional elongation machinery. Although it is clear that there are noncatalytic functions in transcription regulation by MAPK, activity of Hog1 is needed for the recruitment of the kinase onto the promoters and coding regions of osmostress-responsive genes. Recent findings shed light on the link between upstream signalling kinases and direct phosphorylation of histones and/or histone modifiers (reviewed in [76]). Moreover, transcriptional stress responses and the chromatin structure are tightly linked [77, 78]. Why regulation of Paf1 by Mpk1 only affects those cell wall stress-activated genes that are under the control of the noncatalytic Mpk1 pathway is another question that remains unsolved. And in these lines, are the Rlm1-dependent genes regulated by the MAPK during transcriptional elongation? 

Last but not least, to what extent regulation of transcriptional elongation will apply to other yeast and mammalian signalling modules will be interesting avenues for future investigation. In any case, it is becoming clear that mechanisms, which regulate transcription, have been preserved from yeast to mammals.
